# Risk factors of transport gap bending deformity in the treatment of critical-size bone defect after bone transport

**DOI:** 10.1186/s12891-022-05852-2

**Published:** 2022-10-08

**Authors:** Abulaiti Abula, Erlin Cheng, Alimujiang Abulaiti, Kai Liu, Yanshi Liu, Peng Ren

**Affiliations:** Department of Trauma and Microreconstructive Surgery, The First Affiliated Hospital of Xinjiang Medical University, Urumqi, 830054 Xinjiang China

**Keywords:** Bone transport, External fixator, Bone defects, Ilizarov technique, Complications

## Abstract

**Background:**

The purpose of this study was to investigate the risk factors of transport gap bending deformity (TGBD) in the treatment of critical-size bone defect (CSBD) after the removal of the external fixator.

**Methods:**

From January 2008 to December 2019, 178 patients with bone defects of the lower extremity caused by infection were treated by bone transport using a unilateral external fixator in our medical institution. TGBD was defined as the bone callus in the distraction area with a deviation to the force line of the femur (> 10°) or tibia (> 12°) after removal of the external fixator. The Association for the Study and Application of the Method of Ilizarov (ASAMI) standard was applied to assess the bone and functional outcomes. After the data were significant by the T-test or Pearson’s Chi-square test was analyzed, odds ratios were calculated using logistic regression tests to describe factors associated with the diagnosis of TGBD.

**Results:**

A total of 178 patients were enrolled in the study, with a mean follow-up time of 28.6 ± 3.82 months. The positive result of the bacteria isolated test was observed in 144 cases (80.9%). The rate of excellent and good in the bone outcomes (excellent/good/fair/poor/failure, 41/108/15/14/0) was 83.7%, and 92.3% in the functional results (excellent/good/fair/poor/failure, 50/98/16/14/0) according to the ASAMI criteria. TGBD after removal of external fixator occurred in twenty-two patients (12.3%), including 6 tibias, and 16 femurs. Age > 45 years, BMI > 25 kg/m^2^, femoral defect, diabetes, osteoporosis, glucocorticoid intake, duration of infection > 24 months, EFT > 9 months, EFI > 1.8 month/cm were associated significantly with a higher incidence of TGBD in the binary logistic regression analysis (*P* < 0.05). The incidence more than 50% was found in patients with femoral defect (76.1%), osteoporosis (72.7%), BMI > 25 kg/m^2^ (69.0%), diabetes (59.5%), glucocorticoid intake (54.7%). In the multivariate logistic regression analyses, the following factors were associated independently with TGBD, including age > 45 years, BMI > 25 kg/m^2^, femoral defect, diabetes, and osteoporosis.

**Conclusions:**

Bone transport using a unilateral external fixator was a safe and practical method in the treatment of CSBD caused by infection. The top five risk factors of TGBD included femoral defect, BMI > 25 kg/m^2^, duration of bone infection > 24 months, age > 45 years, and diabetes. Age > 45 years, BMI > 25 kg/m^2^, femoral defect, osteoporosis, and diabetes were the independent risk factors. The higher incidence of TGBD may be associated with more risk factors.

## Background

Nonunion caused by infection is a common complication after the treatment of the lower extremity open fracture, which needs plenty of medical resources to resolve [1–6]. Particularly, the bone defect caused by infection is one of the challenging musculoskeletal problems in orthopaedic surgery [4, 7, 8]. Based on the Ilizarov technique, bone transport using a unilateral external fixator is a practical method for the management of bone defects in lower limbs. The advantages of this method include simultaneous repair of soft tissue defects, prevention of complications in the donor area, and early weight-bearing walking, compared with the other technique (Masquelet technique, free vascularized fibular grafting, etc.) [2, 9]. However, postoperative complications caused by a long period of bone transport using a unilateral external fixator have also been presented by previous studies, such as pin tract infection, and delayed union [10, 11]. The risk factor and mechanism of transport gap bending deformity (TGBD) after removal of the external fixator have been gradually noticed but rarely reported.

The occurrence of TGBD after removal of the external fixation is usually underestimated as a postoperative complication. Previous articles noted this phenomenon, but attributed TGBD to the outcome of axial deviation, without further investigation [3, 4, 12]. The deformity < 7° after bone transport is considered an excellent/good grade of bone outcome according to the Association for the Study and Application of the Method of Ilizarov (ASAMI) criteria, but deformity > 7° is likely to affect the limb shape and functional recovery. Some published articles presented that critical-size bone defect (> 6 cm) and long duration of bone infection may be the reasons for poor bone outcomes [13, 14]. Via our previous studies [11, 15], the long duration of infection and more previous surgery usually leads to critical-size bone defect (CSBD) after debridement, which needs a long period of bone transport treatment to repair. The longer external fixation time patients experience, the higher incidence of immobilization osteoporosis they may have, which may indirectly contribute to the development of TGBD. Length of limb discrepancy, abnormal gait, and even pathological fractures may be caused by persistent worsening of TGBD. It may lead to the failure of bone transport if not aware of the risk factors of TGBD, which may place a substantial psychological and financial burden on the patient. Therefore, it is of great importance for orthopaedics to know the potential risk factors with TGBD to effectively prevent its occurrence.

The purpose of this study was to investigate the risk factors of TGBD in the treatment of CSBD after bone transport and provide guiding suggestions to deal with potential risks.

## Methods

After receiving the approval of the Ethics Committee of our hospital, the clinical records and consecutive X-ray photographs of patients with CSBD caused by infection were retrospectively analyzed, from January 2008 to December 2019.

### Inclusion and exclusion criteria

Inclusion criteria: critical-size bone defect (> 6 cm) caused by infection of lower limbs; managed by bone transport using unilateral external fixator; 18 to 60 years old; follow-up time > 20 months.

Exclusion criteria: bone defect ≤ 6 cm; managed by other treatment since severe comorbidities; incomplete medical data; follow-up ≤ 20 months; poor compliance.

### Patients’ data

There were 109 males and 69 females included in this study. Demographics and previous treatment records were obtained utilizing the admission questionnaire scale. All patients were treated with single-level or double-level bone transport using a unilateral external fixator (Orthofix Limb Reconstruction System) after radical debridement. Infection was defined as bone defects associated with sinus drainage, along with positive results of deep bacteriological culture, histological biopsy, and radiological and laboratory findings. A defect > 6 cm was considered as the indication for selecting double-level bone transport. The study was conducted in accordance with the Declaration of Helsinki.

### Surgical technique

Firstly, all necrotic bone and soft tissues were removed completely until the “paprika sign" appears at the end of the remaining bone. The bacterial culture and antibiotic sensitivity test were conducted to exudation to instruct the surgeon to apply the antibiotics. Two Schanz screws (4.5 mm threaded) were inserted in the coronal planes of the proximal and distal femur or tibia with the help of a screw sleeve, respectively. The hydroxyapatite-coated screws could be selected for osteoporotic patients. The screws should be parallel to the articular surface of the distal and proximal tibia or perpendicular to the femoral shaft. A unilateral external fixator was installed and the above screws were enabled to be located respectively in the first hole of distal and proximal clamps. The external fixator was adjusted according to the alignment of the femur or tibia, then another two Schanz screws were inserted in the 3rd and 5th hole of the clamps respectively. Two Schanz screws were inserted through the 1st and 4th holes of the transport segment clamp. When double-level bone transport was used, the transport segment clamp was added and inserted the screws using the same method. After the external fixator sliding clamps were assembled to parallel the axis of the femur or tibia, a minimally invasive osteotomy was performed using a Gigli saw with special care to preserve the periosteum. Finally, the skin defect was covered by the designed direct suture with appropriate tension or keystone flap. An X-ray radiograph was arranged on the second postoperative day and sensitive intravenous antibiotics were conducted for three days.

The distraction phase started after a latent period of seven days, with a rate of 0.25 mm/6 h. Pin tract care was conducted daily. Partial weight-bearing walking was managed on the second postoperative day, and walker or crutch walking was on the second postoperative week. Subsequently, radiography, WBC, ESR, and CRP were examined at 1, 3, 6, 9, 12, 18, and 24 months after bone transport.

### Data collection

The demographics were collected, including age, gender, body mass index (BMI = weight (kg) /height (m^2^)), location of bone defect (femur or tibia), comorbidities (such as diabetes, hypertension, and osteoporosis), glucocorticoid intake, and duration of infection.

Postoperative data were documented, including defect size (DS), type of bone transport (single level and double level), bone union time (BUT), external fixation time (EFT), and external fixation index (EFI). The ASAMI criteria were applied to assess the postoperative outcomes. TGBD was defined as the bone callus in the distraction area with a deviation to the force line of the femur (> 10°) or tibia (> 12°) after removal of the external fixator.EFT referred to the time spent before removing the external fixator. EFI is defined as the ratio of EFT (month) to the distraction regenerate length (cm).

### Potential risk factors

Quantitative variables included age, the duration of bone infection, DS, BUT, EFT, and EFI. And gender, body mass index (normal weight = BMI < 25 kg/m^2^, obesity = BMI > 25 kg/m^2^), location of bone defect (femur or tibia), comorbidities such as diabetes (yes or no), osteoporosis (yes or no), and the history of glucocorticoid intake (yes or no), and the type of bone transport (single level and double level) were attributed to the categorical variables.

### Statistical analysis

The rate of TGBD was analyzed and expressed as a percentage of the total individuals. Categorical variables, such as gender (male or female), BMI > 25 kg/m^2^ (yes or no), location of bone defect (femur or tibia), type of bone transport (single-level or double-level), diabetes (yes or no), hypertension (yes or no), osteoporosis (yes or no), and glucocorticoid intake (yes or no), were analyzed by the Pearson's chi-square test or Fisher exact test. Quantitative variables, including age, duration of infection, DS, BUT, EFT, and EFI were described with mean ± standard deviation and analyzed by the T-test.

The variable with a *P*-value of 0.05 or less in the Pearson's chi-square test, Fisher exact test, or T-test was entered in the multivariate logistic regression model to assess the relationship between the explanatory variable and TGBD. The odd ratio provides a 95% confidence interval and *P*-value. Multivariate logistic regression analysis was used for associated risk factor analysis. It was statistically significant that *P* < 0.05.

## Results

A total of 178 patients were enrolled in the study, with a mean follow-up time of 28.6 ± 3.82 months. There were 109 males and 69 females with a mean age of 39.51 ± 7.15 years. The duration of infection was 23.7 ± 7.23 months, with 3.1 previous surgery per patient. The positive result of the bacteria isolated test was observed in 144 cases (80.9%), including 104 patients (72.2%) with S. aureus, 21 patients (14.5%) with P. cuprina, and 19 patients (13.1%) with E. coli. There were 142 tibial bone defects and 36 femoral bone defects, with a mean DS of 6.19 ± 1.31 cm. The mean BUT, EFT, and EFI in this cohort were respectively 8.07 ± 0.53 months, 8.83 ± 0.47 months, and 1.62 ± 0.33 months/cm. The rate of excellent and good in the bone outcomes (excellent/good/fair/poor/failure, 41/108/15/14/0) was 83.7%, and 92.3% in the functional results (excellent/good/fair/poor/failure, 50/98/16/14/0) according to the ASAMI criteria.

**Fig. 1:**
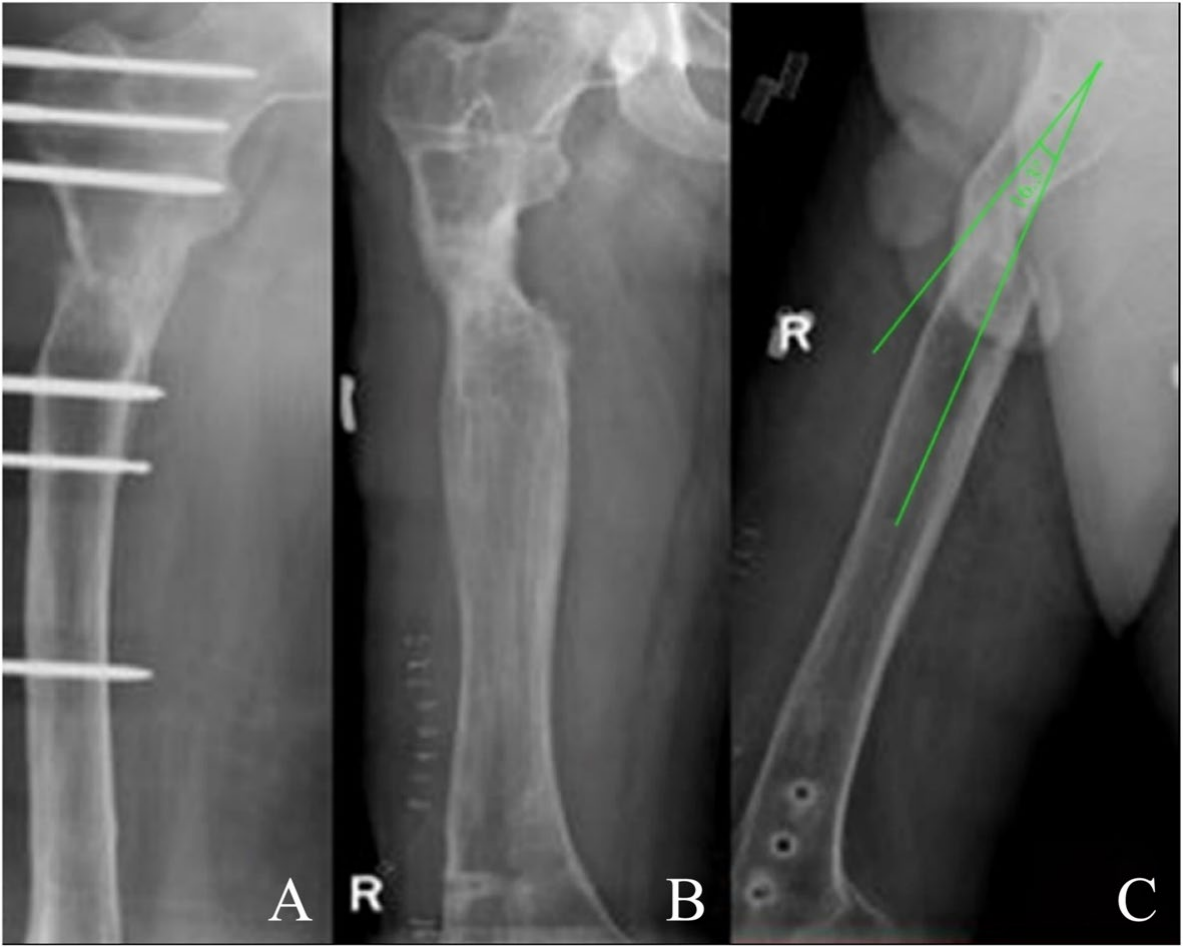
A 39-year-old male patient with right femoral bone defect caused by post-traumatic osteomyelitis was treated by single-level bone transport using a unilateral external fixator. **A** Bone transport was completed with good regenerate consolidation and docking union was achieved at 14th postoperative months. **B**, **C** The TGBD with an offset axial line of force of 16.3° was noticed after removal of the external fixation

**Fig. 2:**
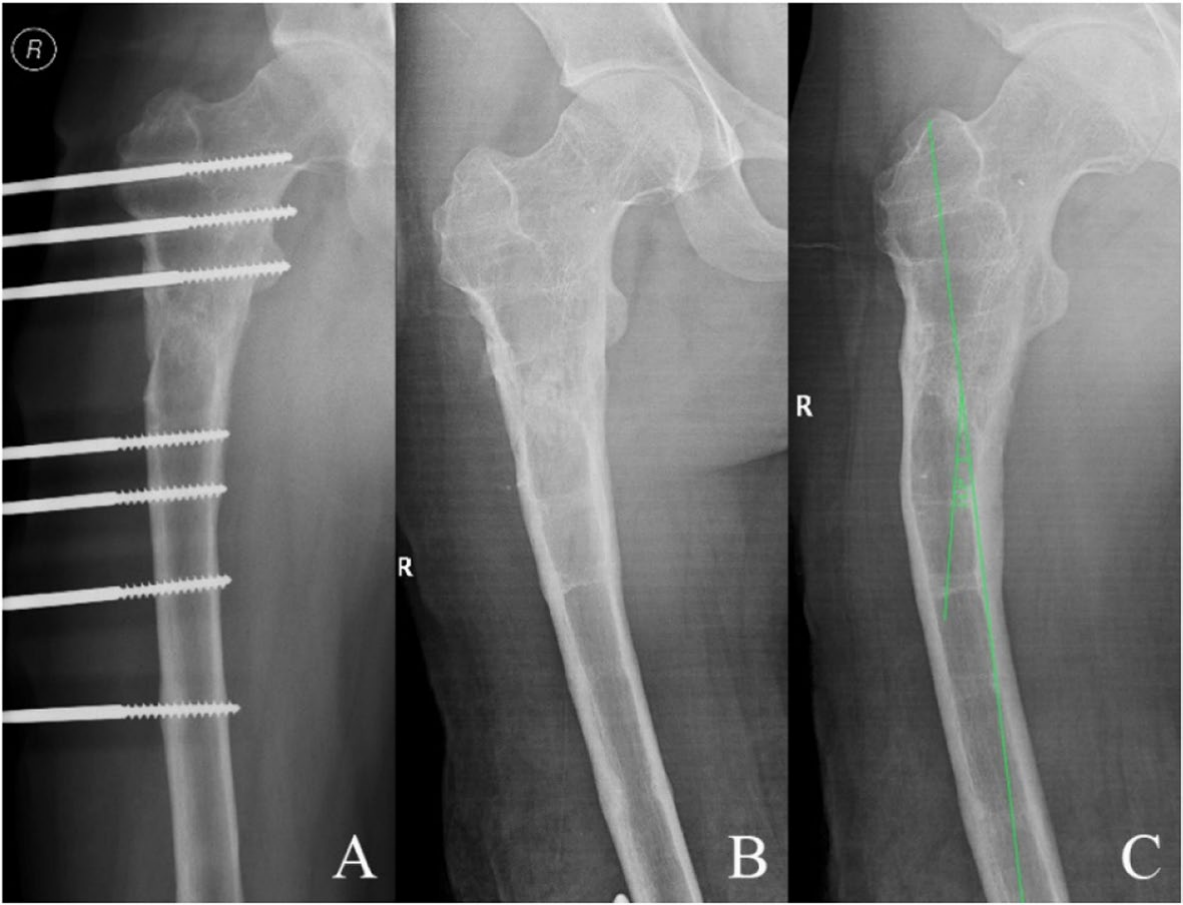
A 47-year-old male patient with right femoral bone defect caused by infection was managed by single-level bone transport. **A** Bone transport was completed with satisfactory consolidation, and docking union was received at 10th postoperative months. **B**/**C** The TGBD with an offset axial line of force of 14.4° was observed after removal the external fixation

**Fig. 3:**
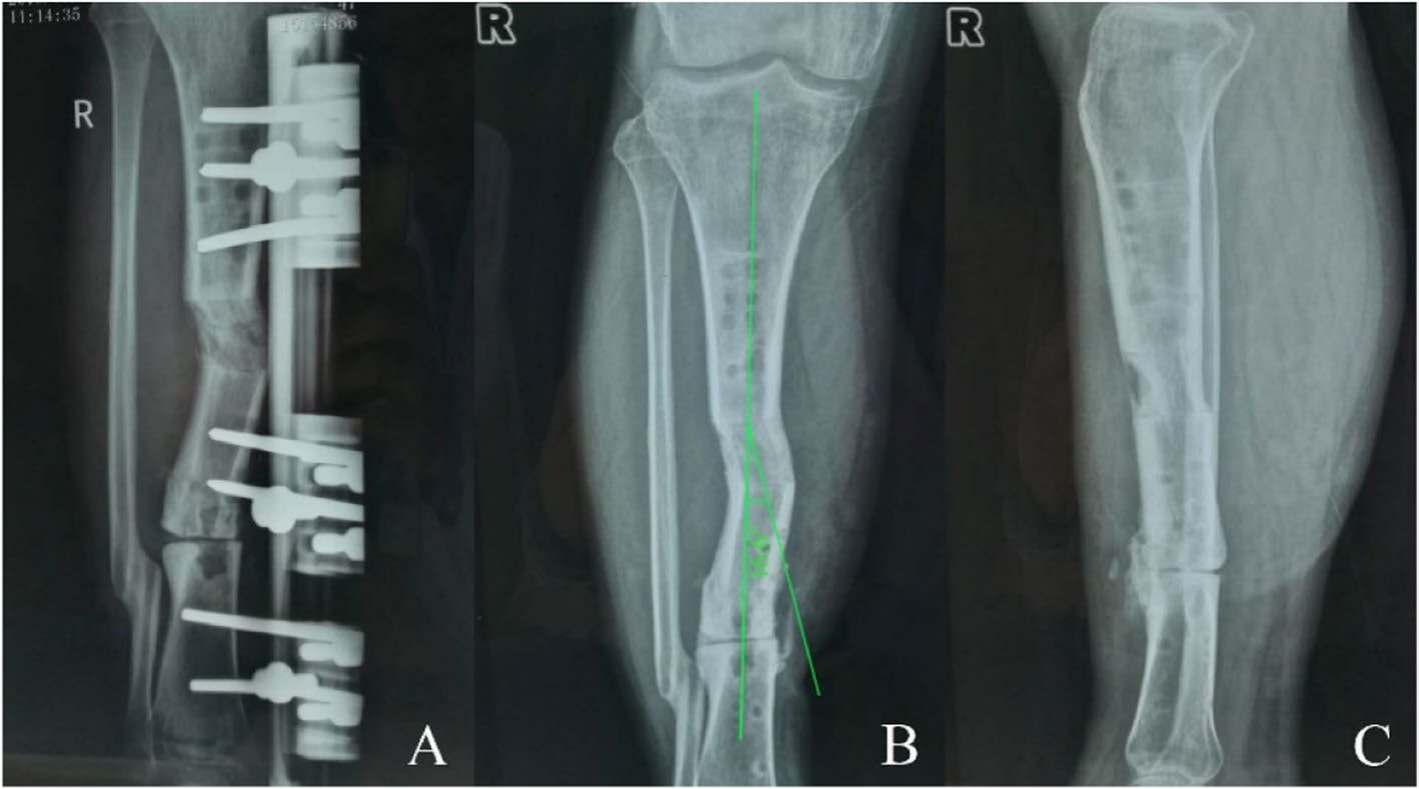
A 41-year-old male patient with right tibial bone defect caused by post-traumatic osteomyelitis was managed by single-level bone transport. **A** Bone transport was completed with satisfactory consolidation and docking union was received at 12th postoperative months. **B**, **C** The TGBD with an offset axial line of force of 16.6° was observed after removal the external fixation

**Fig. 4:**
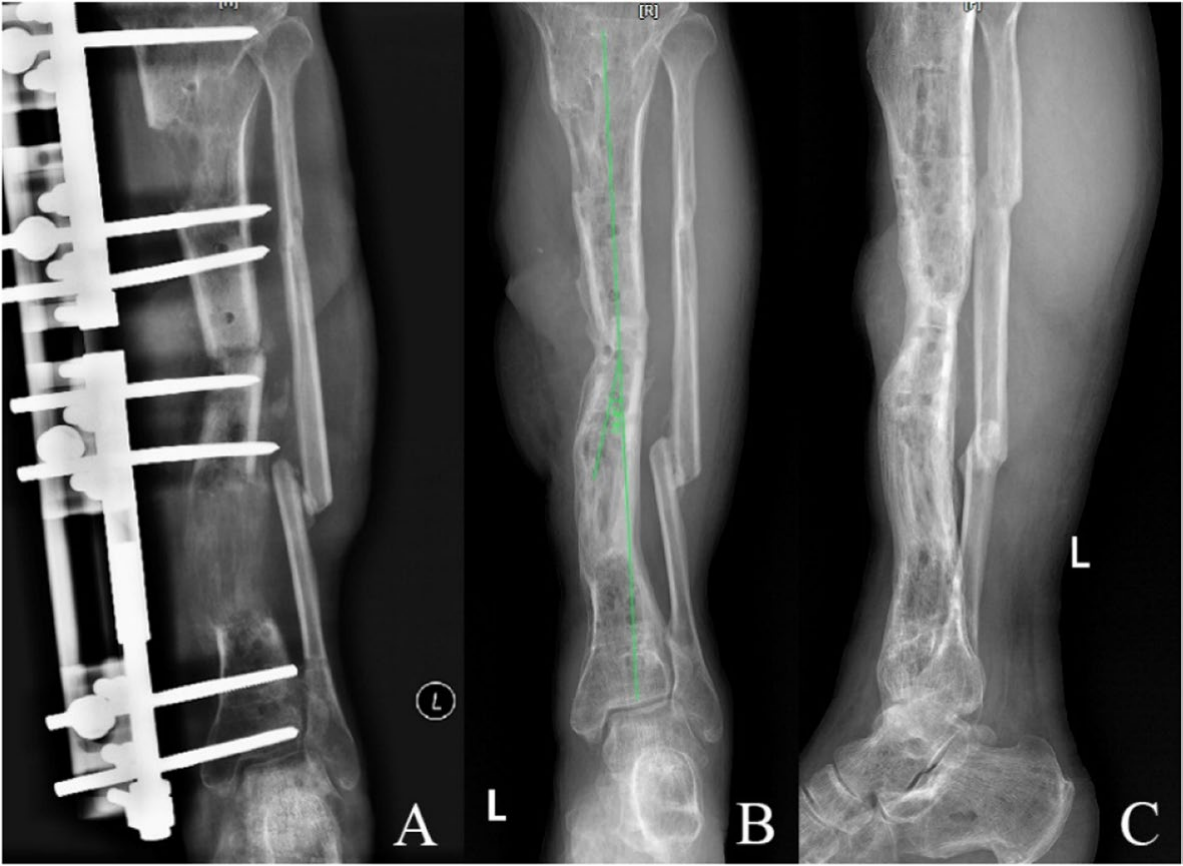
A 42-year-old male patient with left tibial bone defect caused by infection was managed by double-level bone transport. **A** Bone transport was completed with satisfactory consolidation, and docking union was received at 11th postoperative months. **B**/**C** The TGBD with an offset axial line of force of 14.4° was observed after removal the external fixation

TGBD after removal of external fixator occurred in twenty-two patients (12.3%), including 6 tibias, and 16 femurs. The patients were divided into two groups according to the presence or absence of TGBD (Table 1). The statistically significant variables were entered into binary and multiple logistic regression analysis. There was no significant difference concerning gender, type of bone transport, hypertension, DS, and BUT > 9 months (*P* > 0.05). Age > 45 years, BMI > 25 kg/m^2^, femoral defect, diabetes, osteoporosis, glucocorticoid intake, duration of infection > 24 months, EFT > 9 months, EFI > 1.8 month/cm were associated significantly with a higher incidence of TGBD in the binary logistic regression analysis (*P* < 0.05, Table 2). DS > 5 cm was not a significant association with TGBD (*P* > 0.05). The incidence more than 50% was found in patients with femoral defect (76.1%), osteoporosis (72.7%), BMI > 25 kg/m^2^ (69.0%), diabetes (59.5%), glucocorticoid intake (54.7%). In the multivariate logistic regression analyses, the following factors were associated independently with TGBD, including age > 45 years, BMI > 25 kg/m^2^, femoral defect, diabetes, and osteoporosis (Table 3). Regarding the accessorial outcome of bone transport complications, the incidence of TGBD per individual per risk factor was presented (Table 4). The typical cases of the patient with TGBD was shown in Figs. 1, 2, 3 and 4.

**Table 1:** Baseline characteristics of patients

Factor	TGBD	Not TGBD	t / *χ*^2^	*P* value
Male (%)	18(81.8)	91(58.3)	0.740	0.390
Age, mean ± SD (years)	45.58 ± 6.62	38.05 ± 7.61	3.045	< 0.001
BMI (%)			4.315	0.038
< 25 kg/m^2^	7(31.8)	36(23)		
> 25 kg/m^2^	15(68.1)	120(76.9)		
Location of defects (%)			4.233	0.040
Femur	16(76.1)	11(7)		
Tibia	6(23.8)	145(92.9)		
Type (%)			0.201	0.654
single level	12(54.5)	82(52.5)		
double level	10(45.4)	74(47.4)		
Diabetes yes (%)	9(40.9)	58(37.1)	4.813	0.028
Hypertension yes (%)	4(18.1)	39(25)	0.868	0.351
Osteoporosis yes (%)	16(72.7)	49(31.4)	5.064	0.024
Glucocorticoid intake yes (%)	12(54.5)	36(23)	8.176	0.004
Duration of infection, mean ± SD (month)	25 ± 8.02	23.61 ± 7.08	0.818	0.014
DS, mean ± SD (cm)	6.11 ± 1.33	6.23 ± 1.30	0.940	0.340
BUT, mean ± SD (month)	8.6 ± 0.50	7.99 ± 0.58	2.752	< 0.001
EFT, mean ± SD (month)	9.51 ± 0.38	8.73 ± 0.65	3.668	< 0.001
EFI, mean ± SD (month/cm)	1.99 ± 0.28	1.58 ± 0.40	2.912	< 0.001
Follow-up time (months)	28.17 ± 4.29	28.64 ± 3.51	0.681	0.462

*TGBD* transport gap bending deformity, *BMI* body mass index, *BUT* bone union time, *DS* defect size, *EFT* external fixation time, *EFI* external fixation index

**Table 2:** Binary logistic regression analysis of risk factors for TGBD

Factor	Odds ratio (95% CI)	Standard error	*P* value
Age > 45 years	0.88(0.82–0.94)	0.037	0.001
BMI > 25 kg/m^2^	2.42(1.01–5.79)	0.445	0.047
Femoral defect	2.51(1.16–5.42)	0.393	0.019
Diabetes	0.46(0.19–0.80)	0.357	0.010
Osteoporosis	0.40(0.18–0.81)	0.363	0.012
Glucocorticoid intake	0.36(0.17–0.76)	0.380	0.008
Duration of infection > 24 months	1.07(0.99–1.15)	0.036	0.042
DS > 5 cm	1.16(0.80–1.66)	0.186	0.425
BUT > 9 months	1.77(0.71–4.45)	0.468	0.219
EFT > 9 months	0.10(0.03–0.33)	0.594	< 0.001
EFI > 1.8 month/cm	0.06(0.01–0.27)	0.731	< 0.001

*TGBD* transport gap bending deformity, *BMI* body mass index, *BUT* bone union time, *DS* defect size, *EFT* external fixation time, *EFI* external fixation index

**Table 3:** Multivariate logistic regression analysis of risk factors for TGBD

Factor	Odds ratio (95% CI)	Standard error	*P* value
Age > 45 years	1.14(1.04–1.24)	0.044	0.003
BMI > 25 kg/m^2^	2.71(0.66–4.03)	0.715	0.012
Femoral defect	2.92(0.91–4.36)	0.593	0.007
Diabetes	0.30(0.10–0.92)	0.569	0.036
Osteoporosis	0.76(0.25–2.29)	0.562	0.031
Glucocorticoid intake	0.97(0.17–0.76)	0.380	0.683
Duration of infection > 24 months	1.08(1.00–1.18)	0.042	0.440
BUT > 9 months	2.49(0.79–7.83)	0.584	0.118
EFT > 9 months	1.85(0.55–3.95)	0.688	0.276
EFI > 1.8 month/cm	2.75(1.13–3.52)	0.870	0.763

*TGBD* transport gap bending deformity, *BMI* body mass index, *BUT* bone union time, *EFT* external fixation time, *EFI* external fixation index

**Table 4:** Incidence of TGBD according to the number of risk factors present

Risk factors(*n*)^a^	Patients (*n*) per risk factor category	Incidence of re-fracture
1	144	12(8.3%)
2	66	15(22.7%)
3	27	8(29.6%)
4	7	3(42.8%)
5	-	-

*TGBD* transport gap bending deformity

^a^To categorize patients whether at risk or not, the continuous risk factors were dichotomised: age > 45 years vs age < 45 years, BMI > 25 kg/m^2^ vs BMI < 25 kg/m^2^, femur vs tibia, diabetes vs not diabetes, osteoporosis vs not osteoporosis, glucocorticoid intake vs not glucocorticoid intake, duration of infection > 24 months vs duration of infection < 24 months, EFT > 9 months vs EFT < 9 months, EFI > 1.8 month/cm vs EFI < 1.8 month/cm

Patients with TGBD were successfully managed by wedge-shaped osteotomy correction surgery. Besides, pin tract infection occurred in 17 cases, delayed union at the docking site in 6 cases, and axial deviation in 11 cases. Pin tract infections were resolved by local dressing change and oral antibiotic therapy. The axial deviation was corrected by adjusting the external fixator under local anaesthesia. Deep pin tract infection and delayed union at the docking site were resolved by revision surgery.

## Discussion

The Ilizarov technique has been successfully used for many years to treat bone defects caused by pathological diseases [4, 6–8, 16–18]. Bone transport using a unilateral external fixator, based on the Ilizarov technique, has been widely utilized for the treatment of CSBD caused by infection in lower limbs [2, 19]. However, to our knowledge, few studies focus on the occurrence of TGBD after the removal of the external fixator at the end of bone transport. In this study, complete medical records of 178 patients with CSBD managed by bone transport using a unilateral external fixator were collected and analyzed to illustrate the associated risk factors of TGBD. Briefly, TGBD was observed in 22 patients (12.3%). Femoral defect, BMI > 25 kg/m^2^, duration of infection > 24 months, age > 45 years, and diabetes were the top five risk factors. The incidence of developing TGBD among patients having three or more risk factors is 22–42%.

The published study pointed out that fresh bone callus with differentiation ability lived in the transport gap and was easy to be changed its growth direction by the mechanical stimulation of external force [20, 21]. During the treatment of bone transport using a unilateral external fixator, TGBD may have occurred if the distraction velocity and EFT were not adjusted to the condition of callus formation in the distraction gap. Though the unilateral external fixator possesses the advantages of simple instalment and better patient acceptance, the stability of the whole frame is poor. The unfair force line distribution caused by the two-dimensional spatial configuration may also result in the TGBD. Therefore, orthopaedic surgeons need to know the potential risk factors of TGBD when using bone transport to treat critical-size bone defects, which may help to design an individual treatment plan to fit with the velocity of callus formation in the distraction gap.

The occurrence of TGBD may be attributed to the physical function, status of the bone, mechanism and location of the bone defect [2, 3, 22–25]. Our results showed that TGBD may mostly occur in patients aged > 45 years (OR0.88, CI0.82–0.94). Via published studies [26–29], ageing was often considered to be accompanied by the loss of bone calcium, which may result in osteoporosis. BUT and EFT may be inevitably increased by this poor bone quality. Further, long bones in the elder usually possess poor ability to cope with additional forces than the youth, such as bending and rotation. In detail, the extraosseous morphology of the bone in the elder, the internal trabecular structure, and the connective tissue filled around the trabeculae are degraded in quantity and biological activity [30–32]. Hence, prophylactic administration of calcium supplementation is recommended when bone transport is managed for patients aged > 45 years.

Obesity (OR2.42, CI1.01–5.79) and osteoporosis (OR0.40, CI0.18–0.81) are two common diseases with increasing incidence of occurrence. Fat and bone are connected by many pathways to provide a suitable quality of the growth factor for the skeleton metabolism [33]. For, instance, leptin, adiponectin, and insulin/amylin are all associated with this connection [33]. However, excessive body fat (i.e. abdominal fat) may produce inflammatory cytokines, which stimulate bone resorption and reduce bone strength [33–37]. Despite some studies that have shown that the resistance of lower limb bones to deformity can be enhanced by obesity, more evidence holds that obesity may be involved in an increased risk of skeletal disorders. In our cohort, patient with obesity was up to 2.7 times more likely to acquire TGBD than patient with normal weight. This phenomenon may be the result of the overweight loading on the affected limb and abnormal bone metabolism caused by the inflammatory factor pathway of obesity. Then, it is significant to emphasize weight control through a healthy diet and exercise for preventing TGBD during the treatment of bone transport.

The incidence of TGBD occurred in femoral bone transport (OR2.51, CI1.16–5.42) was higher than in the tibia. With the view of anatomy, there is better soft tissue coverage in the femur, which provides a richer blood supply for new bone formation and reduces BUT, EFT, and EFI. However, the greater against forces the alignment force line of the femur is also brought by such abundant muscle (i.e., quadriceps, anterolateral thigh muscle, etc.) attachments, which requires the surgeon to design a more stable external frame structure. In our experience, it is useful to increase the length of the external fixator railway to obtain a long moment. When the length between the proximal/distal clamp and transport segment clamp is greater than the 1.5 times length of one normal clamp, the stability of the external frame can be maintained by adding a new clamp. Besides, the stronger holding force can be received by inserting three Schanz screws on each of the clamps in the metaphysis, the hydroxyapatite-coated screws are also recommended. However, it is practical to insert the screws in the 1st and 4th holes of the clamp for the clamps in the diaphysis, including the transport segment clamp. A higher rate of nonunion and skeletal structure deformity has been observed in tibial bone transport since the poor blood supply and soft tissue coverage [38, 39]. However, whether there is an association between the occurrence of TGBD and the delayed union is unknown. We consider that the occurrence of the delayed union can be reduced by the early reasonable walking exercise with stable external fixation. Non-weight-bearing walking exercise with crutches for 2–4 weeks after removal of the external fixator is a feasible way to prevent bone shortening or TGBD. Gradual resumption of weight-bearing walking is recommended when the radiographs showed the screw holes were filled with new bone.

A causal relation was also noticed in our cohort between TGBD and comorbidities, such as diabetes (OR0.46, CI0.19–0.80). While microvascular and peripheral nerve degeneration is the most common complication of diabetes, the risk of osteoporosis and pathological skeletal condition should also be considered in the treatment of bone defects in diabetic patients [40–43]. The generated bone callus in the distraction area was affected by the unique interactions, given the mechanism of different types of diabetes [41]. The exact mechanism of bone loss in diabetes is still controversial, but the high concentration of glucose can be toxic to osteoblasts [43], which may be a hindrance to the osteogenesis of the distraction area. Besides, serum osteocalcin levels in a patient with diabetes can be suppressed by hyperglycemia, which may weaken the ability of osteoblasts to synthesize osteopontin for bone formation [42]. In this study, there were 11 diabetic patients combined with osteoporosis. Simultaneously, TGBD was observed in these patients. The incidence of TGBD can be increased by diabetes and its associated complications. Therefore, postoperative management was of great importance for diabetic patients to avoid TGBD, including personalized diabetes plans to achieve good glycemic control, and calcium supplementation.

The microarchitecture of the bone (periosteal and trabecular) and the surrounding blood vessels can be destroyed by the prolonged duration of bacterial infection [44, 45]. Bone mineral density may be reduced, resulting in poor bone healing and even bone degeneration. Simultaneously, disused osteoporosis may be caused by discomfort and pain of the affected limb, which increased the difficulty of bone transport and reconstruction surgery. In this study, the duration of bone infection was more than 16 months per patient. The mean duration of infection (OR1.07, CI0.99–1.15) was up to 25 months in patients with TGBD. Besides, EFT > 9 months (OR0.10, CI0.03–0.33) and EFI > 1.8 month/cm (OR0.06, CI0.01–0.27) were also risk factors of TGBD after bone transport. In our opinion, the disused osteoporosis of the affected limb may have occurred in a patient with CSBD caused by a long duration of infection, resulting in greater EFT and EFI. Hence, double-level bone transport after radical debridement is a practical option for the reconstruction of CSBD in lower limbs. The disused osteoporosis may also be avoided effectively by early walking exercise after bone transport surgery.

Although there were 22 patients with TGBD, fortunately, satisfactory bone and function recovery was received after revision surgery. In our study, the excellent and good rate of the bone and function outcomes was 81.5% and 92.3% respectively. Via analysis of the risk factors, we found that femoral defect, BMI > 25 kg/m2, duration of infection > 24 months, age > 45 years, and diabetes are the top five risk factors. The more risk factors patients had, the higher incidence of TGBD they got (Table 4).

There are some limitations to this study. First of all, it was a retrospective study of patients with CSBD caused by infection in a single medical institution, the results should be carefully considered. Secondly, there was a lack of comparative analysis with other treatment methods. The risk factors of TGBD still need a multi-center study with a large sample to provide explicit analysis for a higher level of evidence.

## Conclusion

Bone transport using a unilateral external fixator is a safe and practical method in the treatment of CSBD caused by infection. The top five risk factors of TGBD included femoral defect, BMI > 25 kg/m^2^, duration of infection > 24 months, age > 45 years, and diabetes. Age > 45 years, BMI > 25 kg/m^2^, femoral defect, osteoporosis, and diabetes were the independent risk factors. The higher incidence of TGBD may be associated with more risk factors.

## Data Availability

The datasets analyzed during the current study are available from the corresponding author upon reasonable request.
